# Psychological distress among postpartum women who took opioids during pregnancy: the role of perceived stigma in healthcare settings

**DOI:** 10.1007/s00737-023-01390-5

**Published:** 2023-11-13

**Authors:** Carla M. Bann, Katherine C. Okoniewski, Leslie Clarke, Deanne Wilson-Costello, Stephanie Merhar, Sara DeMauro, Scott Lorch, Namasivayam Ambalavanan, Myriam Peralta-Carcelen, Catherine Limperopoulos, Brenda Poindexter, Jonathan M. Davis, Michele Walsh, Jamie Newman

**Affiliations:** 1 Analytics Division, RTI International, Research Triangle Park, NC USA; 2 Genomics, Ethics, and Translational Research Center, RTI International, Research Triangle Park, NC USA; 3 Department of Pediatrics, Case Western Reserve University, Cleveland, OH USA; 4 Department of Pediatrics, Cincinnati Children’s Hospital Medical Center, Cincinnati, OH USA; 5 Department of Pediatrics, Children’s Hospital of Philadelphia, Philadelphia, PA USA; 6 Division of Neonatology, Children’s Hospital of Philadelphia, Philadelphia, PA USA; 7 Division of Neonatology, University of Alabama at Birmingham, Birmingham, AL USA; 8 Developing Brain Institute, Children’s National Medical Center, Washington, DC USA; 9 Department of Pediatrics, Emory University School of Medicine, Atlanta, GA USA; 10 Department of Pediatrics, Tufts Medical Center, Boston, MA USA; 11 Pregnancy and Perinatology Branch, Eunice Kennedy Shriver National Institute of Child Health and Human Development, Bethesda, MD USA

**Keywords:** Stigma, Mental health, Pregnancy, Opioid-related disorders, Prenatal drug exposure

## Abstract

This study examined the relationship between perceived stigma in healthcare settings during pregnancy and psychological distress and well-being in the postpartum period among individuals who took opioids while pregnant. Analyses included 134 birth mothers of opioid-exposed infants. At 0–1 months postpartum, perceived stigma and psychological distress were measured using the Prenatal Opioid use Perceived Stigma scale and measures from the Patient-Reported Outcome Measurement Information System (PROMIS). Food insecurity, housing instability, and Adverse Childhood Experiences (ACEs) were also assessed. Linear and generalized linear mixed-effect models were conducted to compare PROMIS scale scores and unmet needs by stigma, adjusting for site/location, age, race/ethnicity, marital status, education, public insurance, and parity. More than half of participants (54%) perceived stigma in healthcare settings. Individuals reporting stigma had higher depression, anxiety, and anger scores (*p* < 0.001) indicating greater psychological distress in the postpartum period compared to those reporting no stigma, after controlling for demographic characteristics. In addition, they scored significantly lower on the PROMIS meaning and purpose scale, an indicator of well-being (*p* = 0.002). Those reporting stigma were more likely to have food insecurity (*p* = 0.003), three or more ACEs (*p* = 0.040), verbal or physical abuse during pregnancy (*p* < 0.001), and less emotional support (*p* = 0.006) than those who did not. An association was observed between perceived stigma in the prenatal period and psychological distress in the postpartum period, providing support for stigma reduction interventions and education for healthcare providers on trauma-informed care.

## Introduction

Opioid use disorder (OUD) continues at a higher rate in individuals who identify as women (henceforth referred to as women) than in men within the ongoing opioid epidemic in the United States (Hales et al. 2020). For those in childbearing years, opioid misuse affected approximately 8% of U.S. women in 2018 and 2019 (Substance Abuse and Mental Health Services Administration 2020). Ko et al. (2020) estimated that in 2019, 6.6% of pregnant people in the U.S. used opioids during pregnancy with over 20% of those reporting misuse. Consequentially, Neonatal Opioid Withdrawal Syndrome (NOWS) has also increased over the past several years in the US (Hirai et al. 2021) and in other countries such as Canada (Turner et al. 2015; Plouffe et al. 2023) and Austria (Bauchinger et al. 2015). Beyond the initial neonatal period, NOWS can significantly impact the developmental trajectory of these infants, representing the lasting effect of opioid use during pregnancy (Benninger et al. 2023).

Comorbid mental health disorders are common among pregnant women with OUDs. Specifically, depression and anxiety are present in 25%–33% of pregnant individuals with an OUD (Arnaudo et al. 2017). Women with an OUD are also more likely to have experienced adverse childhood events such as emotional, physical, or sexual abuse (Green et al. 2009), averaging three or more adverse childhood events prior to the age of 18 (Evans et al. 2020; Gannon et al. 2021).

Beyond the prenatal period, it is well established that perinatal depression and anxiety disorders (PMADs) and maternal postpartum mental health have a wide-reaching effect, particularly within the first months of an infant’s life. Experiences of PMADs negatively impact the dyadic relationship between mother and infant, which can compromise the developmental trajectory of infants across behavioral, cognitive, and motor domains (Hoffman et al. 2017; Koutra et al. 2013) .

Other factors such as food insecurity, housing instability, and limited social support are frequently present in pregnant persons in treatment for OUD. Rose-Jacobs et al. (2019) observed that upward of 56% of their U.S.-based sample reported food insecurity, 60% reported housing instability, and 42% reported both. A less frequently examined factor related to mental health and well-being of women with an OUD is healthcare-based stigma. Chaiyachati and Schiff (2023) have suggested that health-related stigma be considered as a social determinant of health, describing it as a bidirectional barrier that impedes patient engagement and provider efficacy, resulting in discriminatory interactions and poor health outcomes. Pregnant women may be particularly susceptible to stigma in healthcare settings given the potential effects of their drug use on their unborn child. Women receiving treatment for an OUD during pregnancy have recalled feelings of judgment and stigma within the healthcare setting which compromises trust and overall care (Cleveland and Bonugli 2014). A pregnant woman may not receive adequate prenatal care during pregnancy as a result of this stigma (Bann et al. 2023), further diminishing care among a subgroup that is already at risk for inadequate prenatal and postpartum care (Simmons and Austin 2022) and thereby resulting in increased risk for both mother and child. However, limited research has been conducted on potential longer-term effects of stigma on women with OUD beyond the immediate healthcare experience.

The current study aims to examine the relationship between perceived stigma in healthcare settings during pregnancy and well-being and mental health in the postpartum period among individuals who took opioids while pregnant. This study also explores unmet needs and negative experiences among those experiencing stigma in healthcare settings.

## Materials and methods

### Study design and participants

This analysis includes 134 birth mothers of infants with antenatal opioid exposure who were enrolled in the Advancing Clinical Trials in Neonatal Opioid Withdrawal Syndrome (ACT NOW) Outcomes of Babies with Opioid Exposure (OBOE) Study. Participants were enrolled if their infant was exposed to opioids in the second or third trimester and if their infant was born at or after 37 weeks gestation. Participants were excluded if they reported heavy alcohol use during pregnancy (eight or more alcoholic drinks per week) or if their infant had known chromosomal or congenital anomalies with the potential to affect the central nervous system, 5-min Apgar score of less than 5, any requirement for positive pressure ventilation in the Neonatal Intensive Care Unit, inability to return for outpatient MRI or follow-up, and intrauterine growth restriction below the third percentile. The OBOE Study protocol is described in detail elsewhere (Bann et al. 2022). Briefly, the OBOE Study is a multi-site U.S.-based prospective longitudinal cohort study of outcomes of infants with antenatal opioid exposure and control (unexposed) infants from birth to 2 years of age. Through a single-Institutional Review Board at Cincinnati Children’s Hospital Medical Center, the OBOE Study clinical sites, neuroimaging core, and data coordinating center received approval for human subjects research activities and informed consent was obtained for all participants.

### Measures

From August 2020 to May 2023, birth mothers of opioid-exposed infants enrolled in the OBOE Study were administered the following questionnaires during the birth hospitalization or during the 0- to 1-month study visit; median (IQR) for time of assessment was 19 (6–31) days following delivery.

#### Well-being and mental health

Select *Parent-Reported Outcome Measure Information System* (PROMIS) (Cella et al. 2010) scales were used to assess well-being and mental health including anxiety (short form 8a), depression (short form 8a), anger (short form 5a), life meaning and purpose (short form 8a), and social support (short form 4a). Low emotional support was defined based on a median split of the PROMIS emotional support scale.

#### Stigma

We used the Prenatal Opioid use Perceived Stigma (POPS) scale to assess perceived stigma. The POPS scale is an eight-item measure specifically designed to assess perceptions of stigma in healthcare settings among individuals who take opioids during pregnancy. The scale assesses three aspects of stigma: delays in care, communication with providers, and patient-provider interactions. For this analysis, the presence of perceived stigma is defined as a yes response to any of the items on the overall scale or individual subscales, as applicable. The POPS scale has demonstrated good reliability (Cronbach’s alpha = 0.88) and construct validity in this population (Bann et al. 2023).

#### Adverse childhood experiences

We administered the *Adverse Childhood Experiences* (ACE) questionnaire to the birth mother, a self-report measure used to capture specific childhood experiences that have been shown to correlate with future social risk factors and negative health outcomes (Felitti et al. 1998) .

#### Housing instability

Mothers were asked whether they spend greater than 50% of the monthly household income on housing.

#### Food insecurity

Mothers screened positive for food insecurity if they responded “often true” or “sometimes true” to either or both questions: During pregnancy, (1) I worried whether my food would run out before I got money to buy more and (2) the food I bought just didn’t last and I didn’t have money to get more (American Academy of Pediatrics, Food Research & Action Center 2021) .

#### Medical history

The study included a maternal medical history form. Of relevance to the current study, data were collected on whether the participant was in an opioid treatment program and types of medication-assisted treatment received. In addition, we collected data on diagnoses of mental health disorders, including depression and anxiety, and related medications.

### Statistical methods

We calculated chi-square tests to compare demographic characteristics of mothers who reported stigma versus no stigma on the POPS scale. To examine the relationship between stigma and mental health, we conducted linear regression models and t-tests to compare mean PROMIS scale scores for depression, anxiety, anger, meaning, and purpose by presence of perceived stigma on the POPS overall scale and subscales. In addition, we conducted logistic regression models to compare the following unmet needs and negative experiences by presence of perceived stigma based on the overall POPS scale: food insecurity, housing instability, three or more ACEs, experiencing verbal or physical abuse during pregnancy, and reporting low emotional support. Linear and logistic regression models controlled for the following demographics: site, maternal age, race/ethnicity, marital status, education, public insurance, and parity. We utilized hot deck imputation to impute missing values for demographic characteristics. All analyses were conducted using SAS version 9.4.

## Results

### Participant characteristics

Demographic characteristics of participants are shown in Table 1. More than a third of participants (35%) were between the ages of 25 and 29; a similar percentage (37%) was between the ages of 30 and 34. The majority of participants were non-Hispanic White race/ethnicity (88%), not married (86%), and had public insurance (90%). Thirty-eight percent of participants had more than a high school education, and 85% were multiparous. Demographic characteristics did not vary significantly by presence of perceived stigma (Table 1).

**Table 1:** Demographic characteristics of participants by presence of stigma (*N* = 134)

Characteristic	All	Stigma	No stigma	*p*
	*N* (%)	*N* (%)	*N* (%)	
Maternal age				
< 25	16 (12)	11 (15)	5 (8)	0.545
25–29	46 (35)	22 (31)	24 (39)	
30–34	49 (37)	27 (38)	22 (36)	
≥ 35	22 (17)	12 (17)	10 (16)	
Non-Hispanic White race/ethnicity				
Yes	118 (88)	65 (90)	53 (85)	0.394
No	16 (12)	7 (10)	9 (15)	
Marital status				
Married	18 (14)	12 (17)	6 (11)	0.303
Not married	110 (86)	59 (83)	51 (89)	
Education				
Less than high school	30 (23)	12 (17)	18 (29)	0.158
High school diploma	52 (39)	27 (39)	25 (40)	
More than high school	50 (38)	31 (44)	19 (31)	
Public insurance				
Yes	120 (90)	66 (93)	54 (87)	0.256
No	13 (10)	5 (7)	8 (13)	
Parity				
1	20 (15)	11 (15)	9 (15)	0.902
2 +	114 (85)	61 (85)	53 (85)	

Perceived stigma is based on the overall POPS scale. Percentages are computed out of the number with data for the corresponding variable. The following variables have cases with missing data: maternal age (*n* = 1), marital status (*n* = 6), education (*n* = 2), and public insurance (*n* = 1)

With respect to treatment, the majority (*N* = 122; 91%) of the mothers in the study had participated in an opioid treatment program at some point during their pregnancy. Among those who received treatment, all were prescribed medication as follows with some participants receiving more than one medication: Buprenorphine (*N* = 69; 57%), Buprenorphine and Naloxone (*N* = 26; 21%), Methadone (*N* = 33; 27%), and Other (*N* = 1; 1%).

### Relationship between stigma and psychological distress

Seventy-two mothers (54%) perceived stigma in healthcare settings during pregnancy (Table 2). Based on the overall POPS scale, mothers who perceived stigma had significantly higher depression scores (Mean (SD) = 51.47 (9.99)) than those who did not perceive stigma (Mean (SD) = 45.82 (8.41); *p* < 0.001). Those who perceived stigma also reported significantly greater anxiety (Mean (SD) = 57.28 (10.99) vs. 49.50 (9.48); *p* < 0.001) and anger (Mean (SD) = 54.85 (11.08) vs. 48.32 (9.39); *p* < 0.001). In addition, positive well-being as measured by the PROMIS Meaning and Purpose Scale was lower among mothers who perceived stigma than those who did not perceive stigma (Mean (SD) = 57.14 (10.26) vs. 61.61 (7.15), *p* = 0.005). Similar patterns were seen for all subscales except for the comparison of meaning and purpose by stigma related to delays in care.

**Table 2:** Mean PROMIS scores by presence of perceived stigma based on POPS scale

Type of stigma	*N*	Depression	Anxiety	Anger	Meaning & purpose
	(Col %)	M (SD)	M (SD)	M (SD)	M (SD)
Overall POPS scale					
Stigma	72 (54)	51.47 (9.99)***	57.28 (10.99)***	54.85 (11.08)***	57.14 (10.26)**
No stigma	62 (46)	45.82 (8.41)	49.50 (9.48)	48.32 (9.39)	61.61 (7.15)
Subscales					
Delays in care					
Stigma	46 (34)	51.50 (9.96)*	56.85 (11.31)*	55.32 (10.97)**	58.05 (9.78)
No stigma	88 (66)	47.45 (9.28)	51.96 (10.49)	49.95 (10.28)	59.84 (8.86)
Communication with providers					
Stigma	58 (44)	53.01 (10.00)***	58.02 (11.20)***	56.10 (11.49)***	56.00 (10.68)***
No stigma	75 (56)	45.71 (8.23)	50.43 (9.72)	48.56 (9.11)	61.71 (7.08)
Patient-provider interaction					
Stigma	50 (37)	53.17 (10.34)***	59.46 (10.38)***	57.14 (11.80)***	54.60 (10.76)***
No stigma	84 (63)	46.31 (8.33)	50.26 (9.91)	48.69 (8.84)	61.92 (6.89)

* *p* < 0.05, ** *p* < 0.01, *** *p* < 0.001; One participant had missing data for communication with providers subscale

**Fig. 1:**
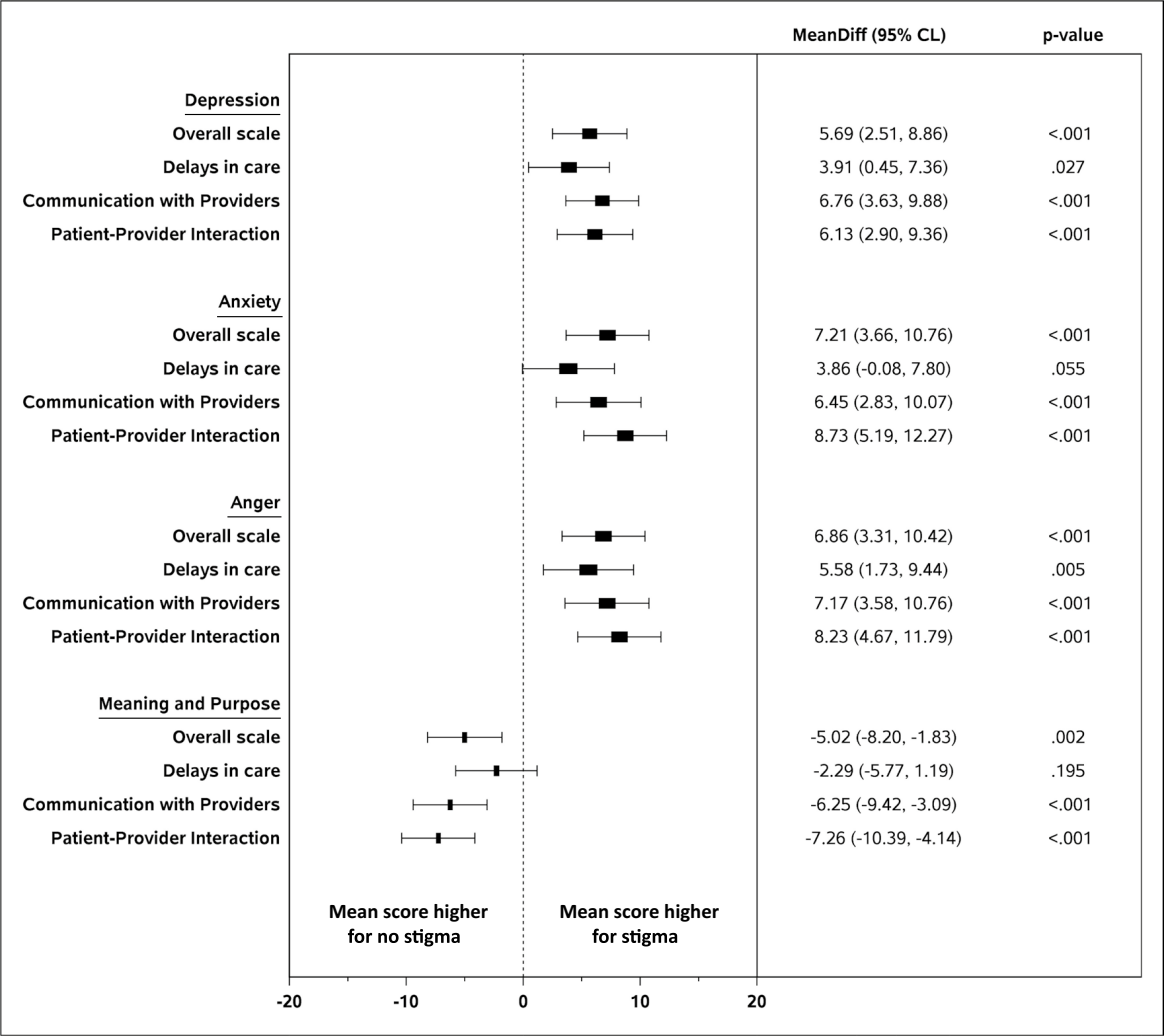
Adjusted mean differences and 95% CL in PROMIS scores by presence of perceived stigma based on POPS overall scale and subscales. Note: Mean differences are adjusted for site, age, race/ethnicity, marital status, education, public insurance, and parity

After controlling for demographics, those reporting stigma had significantly higher scores for depression, anxiety, and anger (*p* < 0.001) (Fig. 1). In addition, they had significantly lower scores on the PROMIS meaning and purpose scale, an indicator of well-being (*p* = 0.013). This pattern was consistent for the POPS communication with providers and patient-provider interaction subscales (Fig. 1). However, while stigma related to delays in care was associated with significantly higher depression (*p* = 0.027) and anger scores (*p* = 0.005), it was not significantly related to anxiety (*p* = 0.055) or meaning and purpose (*p* = 0.195).

### Unmet needs and negative experiences by presence of perceived stigma

**Fig. 2:**
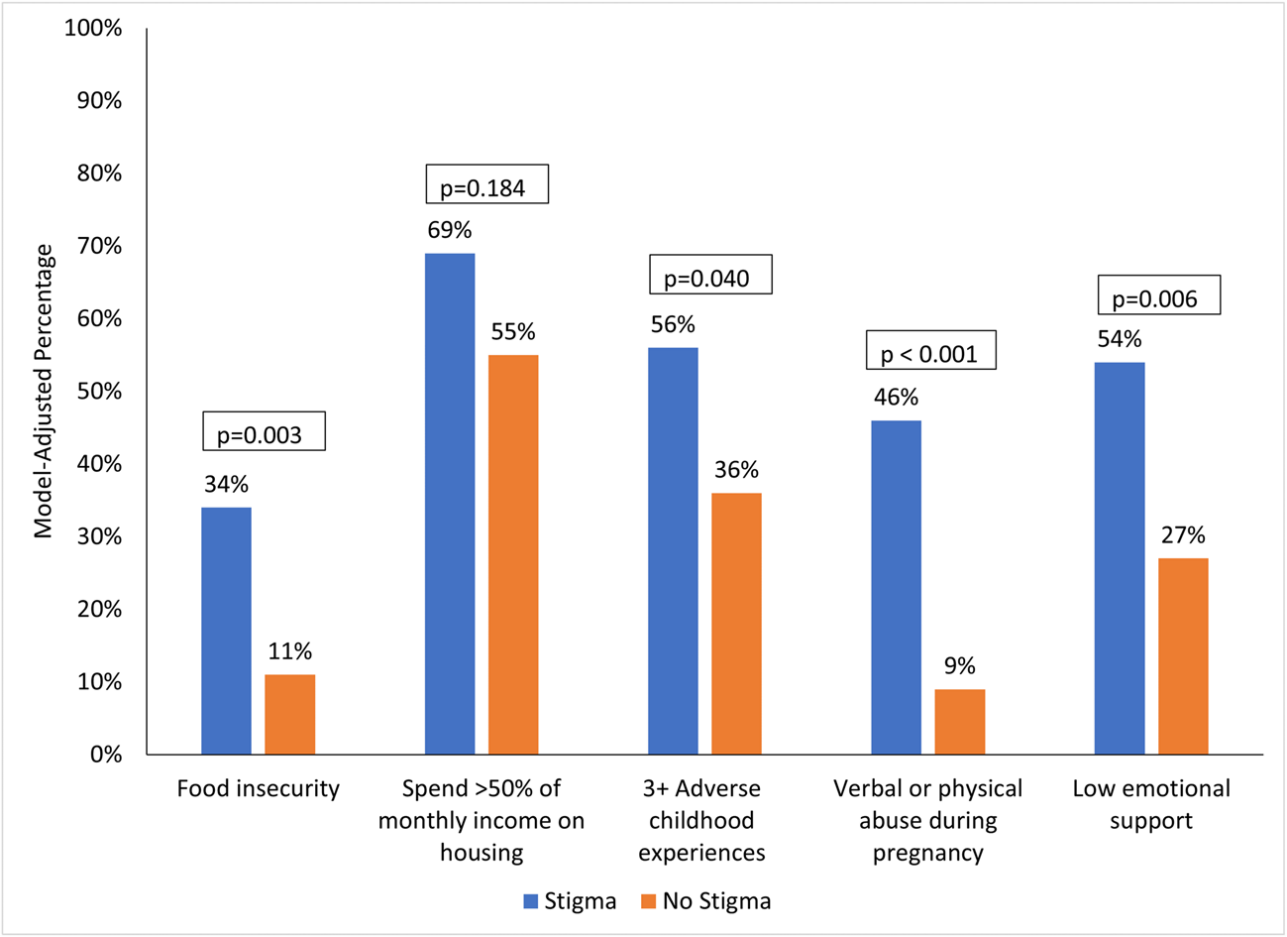
Model-adjusted percentages for unmet needs and negative experiences by presence of stigma. Note: Percentages are adjusted for site, age, non-Hispanic White race/ethnicity, marital status, education, public insurance, and parity

After controlling for demographics, those reporting stigma had more unmet needs and negative experiences (Fig. 2). Thirty-four percent had food insecurity compared to 11% of those without perceived stigma (adjusted odds ratio (AOR) (95% CI) = 4.13 (1.63, 10.49), *p* = 0.003). Similarly, those with stigma compared to those without were more likely to have experienced traumatic events such as three or more ACEs (56% vs. 36%; AOR (95% CI) = 2.31 (1.04, 5.12), p = 0.040) or verbal or physical abuse during pregnancy (46% vs. 9%; AOR (95% CI) = 8.12 (3.04, 21.72), *p* < 0.001). Furthermore, those with perceived stigma were more likely to have poor social support (54% vs. 27%; AOR (95% CI) = 3.17 (1.40, 7.15), *p* = 0.006). No significant differences were observed for spending excessive amounts of income on housing (AOR (95% CI) = 1.82 (0.75, 4.37, *p* = 0.184).

Although those experiencing stigma exhibited greater depression and anxiety based on the PROMIS scale, they were not more likely to have been diagnosed with those conditions (AOR (95% CI) = 1.10 (0.49, 2.48), *p* = 0.822) or to have been prescribed medications for depression or anxiety during pregnancy (AOR (95% CI) = 0.54 (0.23, 1.25), *p* = 0.148).

## Discussion

This study of mothers with opioid use disorder found that those who perceived stigma in healthcare settings during pregnancy had poorer mental health in the immediate postpartum period. Specifically, mothers reporting stigma had more symptoms related to depression and anxiety and expressed greater anger within the month following birth than mothers who did not report stigma. These relationships were strongest for stigma related to communication with providers and patient-provider interactions. In addition to negative symptomatology, stigma was inversely associated with positive well-being measured as feelings of meaning and purpose.

These findings are consistent with research on other stigmatized identities and conditions, such as HIV, weight, lung cancer, and homelessness, which have found an association between stigma and poor mental health outcomes (Alimoradi et al. 2020; Emmer et al. 2019; Gonzalez and Jacobsen 2012; Logie et al. 2019). While extensive research has noted co-morbidities of substance use and mental health (Hunt et al. 2016, 2020; Lai et al. 2015), limited research has been conducted on the potential impact of stigma on mental health among women with substance use disorder, particularly during pregnancy and the post-partum period. Matsumoto et al. (2021) examined the relationship of devaluation stigma and discrimination with mental health among women in publicly funded substance abuse treatment. They found that women who experienced more discrimination, sometimes referred to as “enacted stigma” (Van Brakel 2006) , had poorer mental health symptomatology; however, they did not find a significant relationship between mental health and devaluation stigma attributed to substance use. In contrast to the current study, their research did not focus specifically on stigma related to health care in the perinatal period, suggesting that perhaps stigma may have a greater impact among pregnant and postpartum women who are subject to stigma both due to their substance use and what may be perceived by health care providers as failing to meet the “good mother” ideal (Nichols et al. 2021).

The results of this study suggest that the impact of stigma may extend beyond the immediate interaction with providers in a health care setting. Prior research suggests that perceived public stigma can lead to internalized stigma (i.e., self-stigma) (Vogel et al. 2013). It is possible that women in the current study who perceived stigma and experienced negative interactions with health care providers may have internalized those beliefs which in turn impacted their mental health. Depression in pregnancy is a significant risk factor for postpartum depression (Liu et al. 2022), underscoring the importance of screening for and treating depression during pregnancy. In addition, a study of women with public insurance found that mental health was associated with opioid overdose deaths in the postpartum period (Suarez et al. 2023). Further research is needed to fully understand the relationship between stigma and longer-term outcomes among pregnant women with opioid use disorder.

Participants in this study experienced adverse childhood events at a rate of three or greater across the lifespan, as seen in other investigations within this population (Evans et al. 2020; Gannon et al. 2021). Mothers reporting stigma were more likely to have experienced trauma in childhood or pregnancy and to have other unmet needs. These results suggest that the mothers who are in greatest need of help may be most likely to encounter stigma as a roadblock to receiving that help. Furthermore, in this study, mothers experiencing stigma were less likely to have emotional support from family and friends, possibly resulting in greater dependence on health care providers for help in addressing mental health concerns, accessing substance use treatment, and obtaining other needed resources, as well as potentially deepening the negative impact when this type of help and support is withheld.

For women with OUD, pregnancy provides an opportunity for health care providers to intervene to improve maternal and infant outcomes. Research suggests that pregnancy is a time when women with substance use disorder have greater motivation to obtain treatment (Jackson and Shannon 2012; Mitchell et al. 2008). However, pregnant women with OUD may avoid health care due to fear of detection (Stone 2015) and when venturing into a health care setting, they may encounter a myriad of stigmatizing beliefs from providers (Nichols et al. 2021) similar to the stigma reported by women in the current study. Even mothers with OUD who are using medication-assisted treatment consistent with clinical guidelines (SAMHSA 2018) may encounter stigma attached to use of agonist medications (Witte et al. 2021).

Further research is needed on interventions to reduce stigma in health care settings and improve mental health for pregnant and postpartum women with opioid use disorder. The current and past trauma experienced by mothers in this study emphasizes the importance of implementing trauma-informed care with this population across specialty areas and professionals. Involving both providers and women with lived experience in the design of these interventions could help to improve communication and understanding between the two groups. For example, it may be helpful for providers to understand that women who have experienced trauma may have developed certain strategies and behaviors to survive those situations (Weber et al. 2021). Overall, improved understanding that a multiplicative profile of factors intersects and run parallel to perceived stigma can allow for more targeted intervention services.

## Limitations

A limitation of the study is the assessment of stigma and psychological distress at the same time point. However, the questions related to stigma specifically referred to the pregnancy period and were asked soon after pregnancy ended (i.e., birth of the baby) and therefore, recall bias should be minimized. Also, as previously discussed by Bann et al. (2023), infants enrolled in the OBOE study as per inclusion criteria were medically stable, were born at term to mothers that did not report heavy alcohol use (defined as eight or more drinks per week) and had families that expressed an ability to return for follow-up visits. It is possible that mothers not meeting these criteria would have reported greater stigma and more unmet needs and negative experiences. Despite efforts to increase racial and ethnic diversity, the participants for this study were largely White, non-Hispanic (88%), which limited exploration of differences in stigma and unmet needs across racial and ethnic groups. Additionally, participants were primarily publicly insured, limiting generalizability across populations.

## Conclusions

This study provides a better understanding of the needs of individuals at the intersection of pregnancy, opioid use, and stigma in healthcare settings. Our findings support the necessity of interventions aimed at reducing stigma and reinforce the need for healthcare provider education on trauma-informed care. These types of interventions may enhance prenatal care and postpartum health and potentially improve infant outcome in the neonatal period and beyond.

## Data Availability

Consistent with NIH guidelines, following study completion, a public use dataset will be deposited in the NICHD Data and Specimen Hub (DASH).
